# Infectivity of gastropod-shed third-stage larvae of *Angiostrongylus vasorum* and *Crenosoma vulpis* to dogs

**DOI:** 10.1186/s13071-021-04802-6

**Published:** 2021-06-07

**Authors:** William Robbins, Gary Conboy, Spencer Greenwood, Roland Schaper

**Affiliations:** 1 Department of Biomedical Sciences, University of Prince Edward Island, Atlantic Veterinary College, 550 University Avenue, Charlottetown, PE C1A 4P3 Canada; 2 Department of Pathology and Microbiology, University of Prince Edward Island, Atlantic Veterinary College, 550 University Avenue, Charlottetown, PE C1A 4P3 Canada; 3 Elanco Animal Health GmbH, 40789 Monheim, Germany

**Keywords:** Helminths, Internal parasites, Transmission, French heartworm, Metastrongyloid, Lungworm, *Limax maximus*, Slug

## Abstract

**Background:**

Metastrongyloid parasites *Angiostrongylus vasorum* and *Crenosoma vulpis* infect wild and domestic canids and are important pathogens in dogs. Recent studies indicate that gastropod intermediate hosts infected with various metastrongyloids spontaneously shed infective third-stage larvae (L3) into the environment via feces and mucus under laboratory conditions. Shed L3 retain motility up to 120 days, but whether they retain infectivity was unknown.

**Methods:**

To assess the infectivity of shed L3, the heart/lungs of six red foxes (*Vulpes vulpes*) were obtained from trappers in Newfoundland, Canada. Lungs were examined for first-stage larvae (L1) by the Baermann technique. A high number of viable *A. vasorum* L1 and a low number of *C. vulpis* L1 were recovered from one fox; these were used to infect naïve laboratory-raised *Limax maximus*. L3 recovered from slugs by artificial digestion were fed to two naïve purpose-bred research beagles (100 L3/dog). L1 shed by these two dogs was used to infect 546 *L. maximus* (2000–10,000 L1/slug). L3 shedding was induced by anesthetizing slugs in soda water and transferring them into warm (45 °C) tap water for at least 8 h. Shed L3 recovered from slugs were aliquoted on romaine lettuce in six-well tissue culture plates (80–500 L3/well) and stored at 16 °C/75% relative humidity. Four naïve research beagles were then exposed to 100 L3/dog from larvae stored for 0, 2, 4, or 8 weeks, respectively, after shedding.

**Results:**

All four dogs began shedding *C. vulpis* L1 by 26–36 days post-infection (PI). All four dogs began shedding *A. vasorum* L1 by 50 days PI.

**Conclusions:**

L3 infectivity for the definitive host was retained in both metastrongyloids, indicating the potential for natural infection in dogs through exposure from environmental contamination. As an additional exposure route, eating or licking plant or other material(s) contaminated with metastrongyloid L3 could dramatically increase the number of dogs at risk of infection from these parasites.

**Graphic Abstract:**

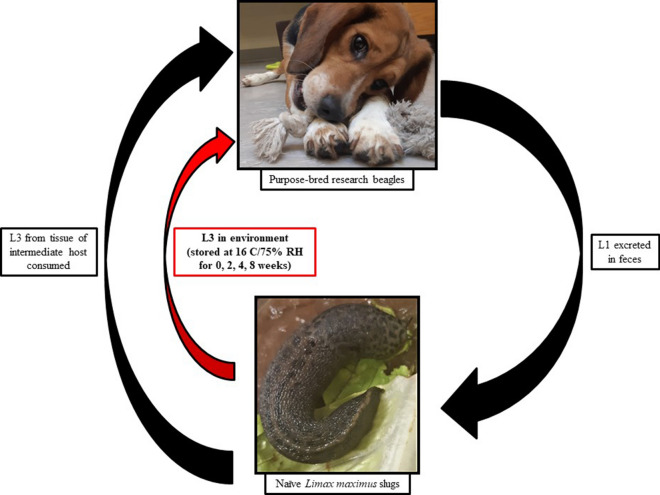

## Background

The superfamily Metastrongyloidea is composed of parasitic nematodes of mammals, most of which are found in the lungs, and some species occur in the frontal sinuses or cardiovascular tissues [1]. Five of the seven Metastrongyloidea families have species requiring a gastropod intermediate host for larval development from the first-stage (L1) to the infective third-stage (L3). Definitive host infection with these metastrongyloids was previously thought to occur solely through the ingestion of L3 contained in the tissues of the gastropod intermediate host or, for some species, a paratenic host [1]. Recent laboratory studies have shown that gastropod shedding of L3 in feces and mucus into the environment is widespread throughout the metastrongyloids. Shedding has been reported in four of the five families containing gastropod-borne species, most prominently in the Protostrongylidae (*Cystocaulus ocreatus*, *Muellerius capillaris*, *Parelaphostrongylus odocoilei*, *Protostrongylus boughtoni*, *Protostrongylus davtyani*, *Protostrongylus kamenskyi*, *Protostrongylus pulmonalis*, *Protostrongylus rufescens*, *Protostrongylus stilesi*, *Protostrongylus tauricus*, *Umingmakstrongylus pallikuukensis*) [2–5]. However, shedding has also been reported in the Angiostrongylidae (*Aelurostrongylus abstrusus*, *Angiostrongylus cantonensis*, *Angiostrongylus costaricensis*, *Angiostrongylus vasorum*), Crenosomatidae (*Crenosoma vulpis*, *Troglostrongylus brevior*, *Troglostrongylus wilsoni*), and Filaroididae (*Oslerus rostratus*) [6–13].

Spontaneous shedding of metastrongyloid L3 has been implicated as a potential transmission route in some cases of *A. cantonensis* infection in humans where the exposure appeared to have been through produce or the handling of gastropods [14–17]. However, direct evidence of natural infection by this route and its epidemiological significance remains unknown. The relative role that environmental contamination due to gastropod shedding may play as a transmission route would correlate with the number of L3 shed and the ability of those larvae to survive and retain infectivity outside of a host for prolonged periods. High levels of shedding (20–100% of the worm burden) and prolonged longevity (6–12 months) have been reported in the Protostrongylidae [4, 5]. Lower levels of shedding (1–3% of the worm burden) have been reported in the families Angiostrongylidae, Crenosomatidae, and Filaroididae [12]. Less is known on the longevity of gastropod-shed L3 in these species. Motile *C. vulpis* L3 maintained outside of a host at 16 °C were recovered up to 120 days later [12]. Whether the L3 retained infectivity for the canid definitive host over this period was not determined.

Crook et al. [8] reported that drowning *A. cantonensis*-infected land snails (*Achatina fulica*) resulted in the release of L3, which retained infectivity when fed 60 h later to the rodent definitive host (*Rattus norvegicus*). Barcante et al. [10] induced shedding of *A. vasorum* L3 from the aquatic snail, *Biomphalaria glabrata*, exposed to 24 h of light and subsequently fed the freshly shed L3 to two dogs resulting in patent infections.

There have been no studies investigating the longevity of metastrongyloid L3 infectivity following shedding from the gastropod intermediate host. This study aimed to determine the infectivity potential and duration of infectivity to the definitive host of gastropod-shed L3 for two important metastrongyloid parasites of dogs, *A. vasorum* and *C. vulpis*.

## Methods

### Parasite source/recovery

The carcasses of six trapped red foxes (*Vulpes vulpes*) were submitted over a 3-week period to the Animal Health Division, Department of Fisheries and Land Resources, Newfoundland and Labrador, Canada. Red foxes were necropsied, and the heart and lungs were then immediately shipped to the Atlantic Veterinary College (AVC) in Charlottetown, Prince Edward Island (PEI), Canada. To determine whether the animals were positive for *A. vasorum*, the right ventricle of the heart was opened, and the pulmonary artery was dissected until adult worms were recovered. The lungs of *A. vasorum*-positive animals were then cut into pieces, which were then subjected to the Baermann technique to recover L1. After approximately 18 h, the fluid from the Baermann technique was collected into 50 ml screw-top test tubes and centrifuged at 700×*g* for 10 min. Following centrifugation, the supernatant was removed and the pellet resuspended in 5 ml of distilled water. The larval recovery was quantified by counting the number of L1 in paired 50 µl (1:100) subsamples. A large number of viable *A. vasorum* L1 were recovered from only one of the foxes, an animal co-infected with *C. vulpis*, and these were used for the study. Although the *C. vulpis* L1 were not detected at the time, the recovered L1 were a mix of the two metastrongyloid species.

### Slug infections

Recovered L1 were counted and evenly distributed into 200 µl subsamples and placed on squares of lettuce (approximately 2 × 2 cm) in six-well tissue culture plates. One laboratory-raised naïve *Limax maximus* slug was then placed into each well containing the lettuce and L1 solution. Tissue culture plates were then placed in environmental chambers (Caron model #6010 and Binder model #KBF 115-UL) set to 16 °C (± 0.2) and 75% RH (± 2%). Once the slugs had consumed the lettuce (and presumably the L1), or after 48 h, they were transferred to larger plastic lid-locked containers, with a maximum of 30 slugs per container, and fed romaine lettuce. L1 recovered from the red fox lung tissue were fed to 40 *L. maximus* (1600 L1/slug); L3 recovered from these slugs were used to infect dogs A and B. L1 recovered from the feces of dogs A and B were used to infect 546 *L. maximus* (2000–10,000 L1/slug); L3 recovered from these slugs were fed once to each of the four experimental dogs (ED-0, ED-2, ED-4, and ED-8) after 0, 2, 4, and 8 weeks of storage, respectively.

### L3 recovery: gastropod digestion

Eight weeks post-infection to the L1 from the red fox, slugs were artificially digested in 50 ml tubes (maximum of 5 g of tissue/tube) for 2 h at 37 °C in a pepsin-HCl solution (50 ml deH_2_O + 0.4 ml HCl + 0.3 g pepsin/slug) to release L3 [12]. After 2 h, slug carcasses were removed from the 50 ml tubes, and the fluid contents were centrifuged at 700×*g* for 10 min. Following centrifugation, the supernatant was removed, and the pellet was resuspended in 2.5 ml of distilled water. Using a dissecting microscope, the L3 were counted, and 100 L3 were placed separately into two 15 ml test tubes. These were fed to dogs A and B (100 L3/dog).

### L3 recovery: water-induced shedding

Third-stage larval shedding was initiated using a modification of the methods used by Cheng and Alicata [6] and Crook et al. [8]. Briefly, after a minimum of 6 weeks post-infection, *L. maximus* were anesthetized in soda water for 15 min (adapted from Cooper [18]). Anesthetized slugs were wrapped in double-layered cheesecloth and transferred to 50 ml screw-top tubes filled with warm (45 °C) tap water for a minimum of 8 h. Slug carcasses were removed and placed a second time into new 50 ml screw-top tubes with warm tap water (45 °C) for a minimum of 8 h, after which the carcasses were discarded. All tubes were centrifuged at 700×*g* for 10 min. The supernatant was removed, the resulting pellet was resuspended in 2.5 ml of distilled water, and the L3 enumerated. The L3 were recovered from 5, 7, 13, and 30 *L. maximus* for each of the storage time periods (0, 2, 4, and 8), respectively.

### Storage of L3

Shed L3 were given immediately after recovery to the zero-week experimental dog (ED-0). Three storage times were also evaluated: 8 weeks, 4 weeks, and 2 weeks. A total of 3820 shed L3 were stored for 8 weeks, 2116 L3 were stored for 4 weeks, and 1098 for 2 weeks (Fig. 1). Larvae were stored on romaine lettuce in six-well tissue culture plates (80–500 L3/well) at 16° C/75% RH.

**Fig. 1:**
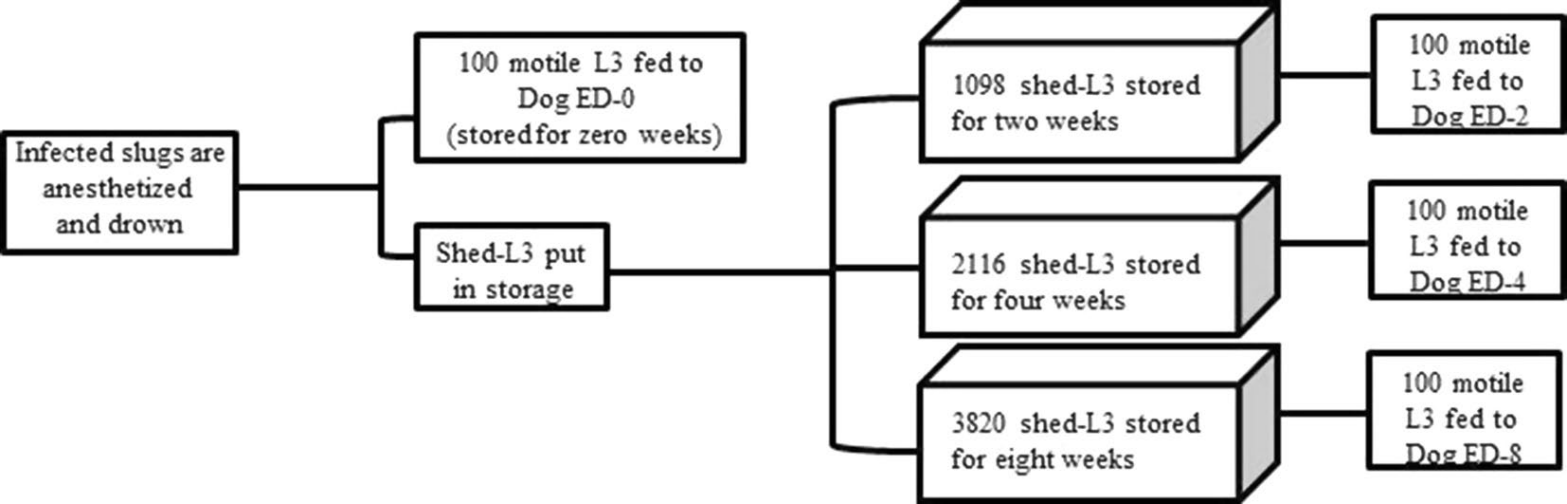
Experimental plan to evaluate the infectivity of gastropod-shed *Angiostrongylus vasorum* and *Crenosoma vulpis* third-stage larvae to dogs

### Recovery of stored (2, 4, and 8 weeks) L3

Following storage, L3 were collected by placing the lettuce and paper towel from each well, wrapped in a double layer of cheesecloth, into 50-ml centrifuge tubes filled with warm digest solution. The L3 were recovered as per the gastropod digestion procedure detailed above. Additionally, L3 were also collected from the six-well plates by adding warm digest solution to each well and examining the plate using a dissecting microscope. Only vigorously motile or tightly coiled L3 were collected for the infection trials target infective dose of 100 L3/dog. Where possible, an additional 100 tightly coiled or actively motile L3 from each storage time period were recovered for morphological evaluation to determine the species composition. These larvae were fixed in hot (65 °C) 2.5% formalin saline.

### Dog infections

A total of six male, purpose-bred research beagles about 9 months of age and weighing between 9 and 11 kg were purchased (Marshall BioResources, North Rose, NY, USA) and brought to the Atlantic Veterinary College (AVC). Two dogs (dogs A and B) were used to supply the large numbers of L1 needed for the infectivity study. The remaining four dogs (ED-0, ED-2, ED-4, and ED-8) were used for the experimental infections using the stored L3. All dogs were examined upon receipt by the University Veterinarian. Fecal samples were examined by ZnSO_4_ centrifugal flotation (S.G. = 1.18) and the Baermann technique [19]. Dogs were acclimated to the housing facility for at least 1 week before L3 exposure. Within the housing room, dogs remained in separate kennels except during socialization times with their respective cohorts (i.e., dogs A and B did not socialize with the experimentally infected dogs ED-0, ED-2, ED-4, ED-8).

The infective dose (100 L3/dog) was administered by pipetting the L3 into two gelatin capsules and giving them to each dog per os. After exposure, dogs remained in their kennels and were monitored every 30 min for 4 h to ensure the capsules were not regurgitated. Following sufficient collection of L1 from dogs A and B, infection, collection, and storage of shed L3, the remaining four purpose-bred naïve research beagles (ED-0, ED-2, ED-4, and ED-8) were each given 100 shed L3 in two gelatin capsules administered as above.

### Diagnostic surveillance

Weekly quantitative Baermann examinations using 12 g of feces were performed beginning at 4 weeks post-infection on all dogs (A, B, 0, 2, 4, and 8) throughout the study. The remainder of the fluid from the weekly Baermann examinations after counting the larvae was hot-fixed in 2.5% formalin saline. The species composition percentage (*A. vasorum* and *C. vulpis*) was determined by morphological identification of 100 L1 from each sample time point. Following the onset of *A. vasorum* patency in dogs A and B, the entire daily fecal deposit production from each dog was collected, and multiple quantitative Baermann examinations using 20 g of feces were conducted over an 11-week period (9–20 weeks PI) to collect as many L1 as possible. Just prior to anthelmintic treatment, dogs A and B were assessed at 32 weeks PI for potential cardiopulmonary damage due to the experimental infections by ultrasound and echocardiogram (ECG) evaluation by the cardiology unit of the Atlantic Veterinary College Teaching Hospital.

### Treatment and aftercare of dogs

Following the collection of sufficient L1 from dogs A and B, and a minimum of two positive *C. vulpis* and *A. vasorum* weekly Baermann examinations from the experimentally infected dogs, all dogs were treated monthly with topical applications of Advantage Multi^®^ 55 (Elanco Animal Health, 10 mg/kg imidacloprid + 2.5 mg/kg moxidectin) for 6 months. During treatment, feces were monitored by Baermann examination for treatment success. Following successful treatment (indicated by negative Baermann results), feces were monitored weekly for another 6 months to ensure efficacious treatment of the dogs and ensure that there was no possibility for release of *A. vasorum* L1 into the environment.

## Results

**Fig. 2:**
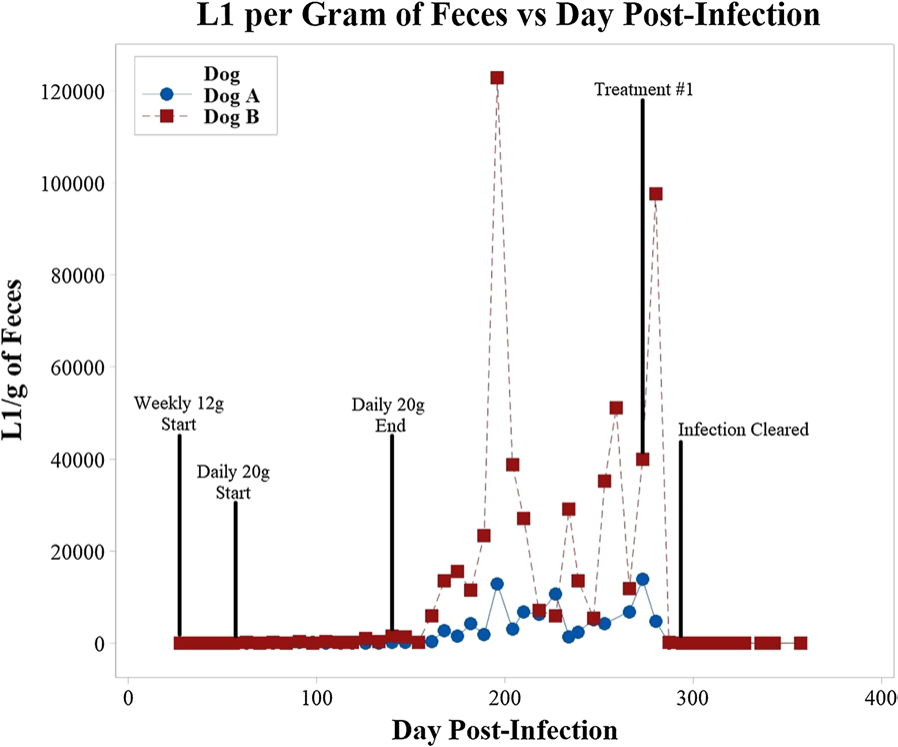
Number of larvae per gram of feces from the weekly 12 g Baermann examinations of dogs A and B over the study period. Both dogs were treated with Advantage Multi^®^ 55 (Elanco Animal Health) on day 273 PI (treatment #1)

*Angiostrongylus vasorum* L1 were recovered from the lung tissue of all six red foxes; however, high numbers of vigorously motile *A. vasorum* L1 were only recovered from one fox. The presence of a low level of *C. vulpis* L1 had not been detected at the time of initial screening in this fox sample, although the possibility of co-infection was considered likely. Both dogs A and B began shedding *C. vulpis* L1 by day 29 and *A. vasorum* L1 by days 56 and 67, respectively. Weekly 12 g Baermann examinations began at 28 days (4 weeks); daily 20 g Baermann examinations began at 63 days (9 weeks), and ended 140 days (20 weeks) post-infection, respectively (Fig. 2). The number of *A. vasorum* L1 collected from both dogs was highly variable, with dog B shedding much higher numbers than dog A (Table 1). Dog B also had a higher percentage of *A. vasorum* L1 (dog B: 99% *A. vasorum*–1% *C. vulpis*; dog A: 97% *A. vasorum*–3% *C. vulpis*), and larval shedding peaked earlier than dog A (dog B: 114 days PI, dog A: 273 days PI) (Table 1). Larval shedding greatly increased for both dogs at approximately 160 days (23 weeks) PI (Fig. 2). From 9–20 weeks PI, all fecal deposits produced by dogs A and B were collected and weighed, allowing an estimate of each dog's total feces production. Using the LPG derived from the weekly 12-g and the daily 20-g quantitative Baermann examinations along with the estimated fecal production, the total estimated number of L1 (*A. vasorum* + *C. vulpis*) shed during the entire period of patency for each dog (29 and 31 weeks) was calculated to be 6,703,506 for dog A and 52,420,293 for dog B (Table 1). After the onset of patency for *A. vasorum*, the species composition of the L1 shed in the feces ranged from 97 to 99% *A. vasorum* and 1–3% *C. vulpis* for both dogs.

**Table 1:** Summary of L1 shedding of *Angiostrongylus vasorum* from experimentally infected dogs A and B

L1 Fecal shedding category	Dog	
	A	B
Mean LPG (SD)	1952 (3485)	11,943 (24,667)
Median LPG (95% CI)	44 (4.3–449)	277 (83–5750)
LPG shedding peak (days PI)	273	114
Median percent *A. vasorum* (95% CI)	97% (95.7–98.0)	99% (98.2–100.0)
Number of L1 collected	287,919	3,706,784
Estimated number of L1 shed during study	6,703,506	52,420,293

Neither dog A nor B exhibited clinical signs at any time during the study, and ultrasound and ECG results were within normal limits. Both dogs were first treated topically with Advantage Multi^®^ 55 (Elanco Animal Health) at 273 days (39 weeks) post-infection. Cessation of larval shedding as determined by Baermann examination occurred at 2 weeks post-treatment and remained negative throughout the entire 6-month monitoring period. Treatments with Advantage Multi^®^ 55 (Elanco Animal Health) continued monthly for an additional 5 months.

A total of 546 *L. maximus* were exposed to the larvae collected from dogs A and B (2000–10,000 L1/slug). Malfunction of one of the environmental chambers resulted in the loss of 225 of the infected slugs. In the surviving slugs, induction of larval shedding was attempted at 4 weeks PI; however, no L3 were recovered at this time. Successful larval shedding in high numbers was induced only from gastropods infected for 6 weeks or more*.* The total number of L3 released from 5 slugs at week 0 were not counted after the infective dose (100 L3) and an additional 100 L3 were collected for morphological examination. A total of 1098 L3 were recovered from 7 slugs for the 2-week (156.9 L3/slug), 2116 L3 from 13 slugs for the 4-week (162.8 L3/slug), and 3820 L3 from 30 slugs for the 8-week storage period (127.3 L3/slug) (Table 2). The species composition of the L3 recovered at week 0 was a mix of 84% *A. vasorum*-16% *C. vulpis* (Table 2). There was a trend of decreasing percentage of *A. vasorum* and increasing *C. vulpis* after storage for 2 and 4 weeks. The number of L3 recovered in excess of the infective dose after storage for 8 weeks was insufficient to allow species determination.

**Table 2:** Percent composition of *Angiostrongylus vasorum* and *Crenosoma vulpis* L3 recovered from slugs following storage times

Storage	Number of slugs	Number of L3	Species mix after storage	
Time	Euthanized	Placed in storage	*A. vasorum* (%)	*C. vulpis* (%)
0 Weeks	5	–*	84	16
2 Weeks	7	1098	68	32
4 Weeks	13	2116	57	43
8 Weeks	30	3820	–	–

*The total number of L3 recovered was not quantified after the infective dose (100 L3) and the larvae needed for morphological identification and hot fixation (100 L3) were collected

Dogs ED-0, ED-2, ED-4, and ED-8 were each exposed to 100 L3 stored for 0, 2, 4, or 8 weeks, respectively. ED-0 began shedding L1 of *C. vulpis* and *A. vasorum* at 36 and 49 days PI, respectively (Table 3). Detection of larval fecal shedding of L1 of *C. vulpis* and *A. vasorum* occurred at 26 and 46 days PI, respectively, in dogs ED-2, ED-4, and ED-8 (Table 3). Mean larvae per gram for both *A. vasorum* and *C. vulpis* generally decreased as storage time increased, except for *C. vulpis* in ED-8 (Table 3). None of the four dogs had any observable clinical signs of infection. Treatment with Advantage Multi^®^ 55 (Elanco Animal Health) began at 7 weeks PI for ED-0 and at 10 weeks PI for ED-2, ED-4, and ED-8. Larvae could no longer be detected on Baermann fecal examination 1 week after treatment and remained negative throughout the entire post-treatment monitoring period. Monthly treatment with Advantage Multi^®^ 55 (Elanco Animal Health) continued for a total of 6 months.

**Table 3:** Duration between exposure to stored *Angiostrongylus vasorum* and *Crenosoma vulpis* L3 and first detection of a patent infection in dogs in addition to the mean larvae per gram (LPG) for both parasites from the weekly 12 g weekly Baermann specimens collected from each dog

Dog	*A. vasorum* shed	*C. vulpis* shed	*n*	Mean (SD) LPG *A. vasorum*	Mean (SD) LPG *C. vulpis*
ED-0	49 days PI	36 days PI	6	16 (31.8)	1.45 (4.36)
ED-2	46 days PI	26 days PI	5	0.141 (0.27)	0.172 (0.29)
ED-4	46 days PI	26 days PI	6	0.013 (0.03)	0.095 (0.23)
ED-8	46 days PI	26 days PI	5	0.0000 (0.00)	8.32 (7.66)

## Discussion

In our previous understanding of parasite transmission of *A. vasorum* and *C. vulpis* to the canid definitive host, animals became infected by the ingestion of L3 contained in the tissues of intermediate and additionally (for *A. vasorum*) paratenic hosts [1, 20]. At no time during the developmental cycle would the L3 be free in the environment. Recent reports of low-level spontaneous shedding of L3 in feces or mucus of gastropods under laboratory conditions suggested the possibility that environmental exposure could play some role in the transmission for these species [12]. The significance of gastropod larval shedding is directly correlated with the ability of the larvae to survive in the environment free from the tissues of a host. The results of this study indicate that L3 held free in the environment under laboratory conditions retained infectivity for up to 8 weeks. The ability of L3 to survive for that length of time outside of a host is inconsistent with the previous view of the transmission of these parasites; if an infection is only acquired by the ingestion of L3 contained in tissues, the larvae would be unlikely to have developed the ability to survive free in the environment. In agreement with previous studies on metastrongyloid larval emergence, the results of this study show that both *A. vasorum* and *C. vulpis* actively exit the gastropod intermediate host [10, 12]. Whether the mechanisms involved in gastropod spontaneous shedding and the water-induced exit of L3 are the same is unknown at this time. The result of the immediate infectivity of these larvae supports the findings of Barcante et al. [10]. Furthermore, the study has shown that *A. vasorum* and *C. vulpis* L3 shed by infected *L. maximus* remain infective to dogs after storage of up to 8 weeks. The survival and retention of infectivity of the L3 kept in relatively warm and humid laboratory conditions (16 °C and 75% RH, respectively) for up to 8 weeks suggests the possibility that environmental contamination with gastropod-shed L3 likely plays a role in natural transmission for both of these metastrongyloid pathogens of dogs. Furthermore, because *Aelurostrongylus abstrusus* (an Angiostrongylid), *Troglostrongylus brevior*, and *Troglostrongylus wilsoni* (crenosomatids) have also been reported to have L3 that spontaneously shed in the feces and mucus of their gastropod intermediate host, it is possible that this is also a potential transmission route for felid metastrongyloids [11, 12]. It is, however, unknown at this time whether felids are able to become infected with these shed L3 [21].

*Angiostrongylus vasorum* can cause fatal infections in dogs, with clinical signs of disease resulting from damage to cardiopulmonary, ocular, and central nervous system tissues; in some cases, infection results in bleeding disorders [22–24]. *Crenosoma vulpis* infection in dogs can result in a non-fatal chronic cough condition [25]. Little is known on the level of infection necessary to induce clinical disease in dogs for either of these pathogenic metastrongyloids. Reported worm burdens associated with fatal *A. vasorum* infection in dogs have ranged from 66 to 572 worms, but in most cases, counts have been in excess of 150 adult worms [26–31]. The experimentally administered infective dose of 100 L3 used in this study resulted in patent infections in dogs A and B, which produced considerable numbers of L1, but neither of the dogs developed clinical disease during the 39 weeks of infection. Based on the cardiology assessment just prior to treatment and the absence of health issues in the 22 months since the dogs were treated, there are no indications that either animal incurred serious permanent cardiopulmonary injury as a result of the infection. Reflecting differences in experimental design and objectives, there is a wide range of *A. vasorum* infective dose levels administered to dogs reported in the literature. Infective dose levels have ranged from as low as 25 to as high as 2800 L3/dog [20, 32]. Experimental infections resulting in clinical disease or death of the animal have been reported with infective doses of 200–2800 L3/dog [20, 33–36]. Although based on a small number of animals, the infective dose given in this study (100 L3/dog) may be a useful target exposure level for utilization in non-terminal investigations or studies of long duration.

The presence of *C. vulpis* L1 was not detected in the original red fox source for *A. vasorum* larvae. The L3 recovered from gastropods exposed to these L1 lead to co-infections in dogs A and B producing fecal larval shedding consisting of a mix of 1–3% *C. vulpis* and 97–99% *A. vasorum*. The L3 recovered from the gastropods exposed to these L1, which were fed fresh to dog ED-0, were a mix of 16% *C. vulpis* and 84% *A. vasorum*. The apparent trend of increasing levels with each passage through the gastropods suggests that *L. maximus* may be a more suitable intermediate host for *C. vulpis* than it is for *A. vasorum*. Alternatively, the developing *C. vulpis* larvae may have some unknown competitive advantage occurring at the expense of the development of the *A. vasorum* larvae in cases of co-infection. Another crenosomatid, *T. brevior*, has been found to have a competitive advantage, where their larvae are able to develop in hibernating snails, whereas the Angiostrongylid, *A. abstrusus*, was not [11, 37]. Furthermore, Giannelli et al. [11] hypothesized that there may be competition between *T. brevior* and *A. abstrusus*, furthering the initial hypothesis of there being a potential for competition between *A. vasorum* and *C. vulpis*. Alterations of epidemiology, disease dynamics, and virulence-associated with co-infections have been reported [38]. There have been few studies on metastrongyloid co-infections in the gastropod intermediate host. Pereira et al. [39] showed an increase in susceptibility to *Schistosoma mansoni* with the simultaneous co-infection with *A. vasorum*. Bonfim et al. [40] found that a co-infection of *Angiostrongylus cantonensis* and *Echinostoma paraensei* resulted in a reduction in the number of recovered L3. Competition for space and resources has been implicated as a potential reason as to why co-infections alter disease dynamics [38, 41–44].

In previous single species experimental infections using our infection model, L3 of *A. vasorum* and *C. vulpis* could be recovered by artificial digestion from *L. maximus* at 4 weeks PI [12]. However, in this study, no water-induced L3 were recovered from *L. maximus* at 4 weeks but were recovered in high numbers at 6 weeks or more PI. This is consistent with the reported timeline for spontaneous shedding of *A. vasorum* L3 from experimentally infected *L. maximus,* which began 20 days PI but peaked at 5 weeks PI [12]. Whether the L3 were present at 4 weeks but incapable of exiting the slug tissues at this time or development of the larvae was delayed due to the co-infection remains unknown.

The number of viable larvae recovered decreased as storage time increased, and there was a trend toward a lowering of larval fecal shedding counts in the dogs with increased storage time. In general, as storage time increased, the number of L1 shed by the infected dogs decreased for both *A. vasorum* and *C. vulpis*. This result was likely due to a reduction of infectivity as storage time increased. The exception to this result was ED-8, which had a higher level of *C. vulpis* fecal larval shedding than the other dogs. This could be due to a possible survival difference between the two parasites. As shown in Table 2, the percentage of viable *A. vasorum* decreases over time, and thus the percentage of viable *C. vulpis* increases. This suggests *C. vulpis* may be able to survive outside of the intermediate host longer than *A. vasorum*.

Within the scope of co-infections, this study has also shown the *L. maximus* slugs are capable of supporting and delivering a co-infective dose to dogs. This represents the first report of co-infected *L. maximus* slugs delivering a co-infective dose to the definitive host. Both *A. vasorum* and *C. vulpis* naturally occur together in many areas, but in North America, they are found together in Newfoundland and, more recently, Nova Scotia [45, 46]. co-infection with both metastrongyloids is common in the red fox population in Newfoundland, and therefore co-infection of the slugs feeding on the feces from these animals is likely also common [45].

The results from this study provide further information surrounding the emergence of metastrongyloid larvae from the intermediate host. The L3 larvae of both *A. vasorum* and *C. vulpis* arising from the gastropod intermediate host were found to be immediately infective (similar to Barcante et al. [10]) and remain infective for up to 8 weeks in storage. Although this study does not confirm this alternative transmission route happens in nature, these results suggest a likelihood of natural transmission occurring through exposure to L3 from environmental contamination through such common activities as ingestion or licking grass or other plant material. This greatly increases the population of dogs at risk of infection of these important metastrongyloid pathogens.

## Conclusions

This study has shown that shed *A. vasorum* and *C. vulpis* L3 survive and remain infective for up to 8 weeks outside of the intermediate host. Our results also indicate a possible survivability difference between *A. vasorum* and *C. vulpis*. Additionally, co-infections of *A. vasorum* and *C. vulpis* are possible in *L. maximus* and can be successfully delivered as a co-infective dose. Finally, these results suggest that exposure through environmental contamination may contribute to natural infections in dogs. As an exposure route, eating or licking plant or other contaminated material(s) could dramatically increase the number of dogs at risk of infection from these parasites.

## Data Availability

Not applicable.

## Abbreviations

AVC: Atlantic Veterinary College
ECG: Echocardiogram
L1: First-stage larvae
L3: Third-stage larvae
LPG: Larvae per gram of feces
PI: Post-infection
PEI: Prince Edward Island
RH: Relative humidity
CCAC: Canadian Council of Animal Care
